# Etiology and Features of Eyes with Rubeosis Iridis among Korean Patients: A Population-Based Single Center Study

**DOI:** 10.1371/journal.pone.0160662

**Published:** 2016-08-04

**Authors:** Yun Cheol Jeong, Young Hoon Hwang

**Affiliations:** Department of Ophthalmology, Konyang University, Kim’s Eye Hospital, Myung-Gok Eye Research Institute, Seoul, Korea; Charité University Medicine Berlin, GERMANY

## Abstract

**Purpose:**

To estimate the etiology and features of the eyes with rubeosis iridis among Korean patients.

**Materials and Methods:**

This study is a retrospective review of 533 Korean patients with rubeosis iridis who visited an eye hospital in Seoul, Korea. We defined rubeosis iridis as visible blood vessels on the iris surface detected during a slit-lamp examination. All cases were reviewed for age at the time of diagnosis, medical history, the most likely cause of rubeosis iridis, visual acuity, and intraocular pressure.

**Results:**

The most commonly observed cause of rubeosis iridis was diabetic retinopathy (DR; n = 337, 63.2%), followed by retinal vein occlusion (RVO; n = 101, 18.9%), ocular ischemic syndrome (OIS; n = 24, 4.5%), retinal detachment (n = 17, 3.2%), and uveitis (n = 15, 2.8%). The cause was classified as miscellaneous in 18 cases (3.4%); in 21 eyes (3.9%), the cause was not clear. Age at the time of rubeosis iridis diagnosis was lower in patients with DR (56.5 years) than in those with RVO (61.0 years) and OIS (64.8 years; P < 0.01). Intraocular pressure of the eyes with DR (37.3 mmHg) and RVO (39.5 mmHg) was higher than that of the eyes with OIS (25.8 mmHg; P < 0.01).

**Conclusion:**

In our population-based single center study, DR was the leading cause of rubeosis iridis followed by RVO and OIS among Korean patients. The clinical characteristics of the eyes with rubeosis iridis differed according to etiology. This finding may be useful when assessing eyes with rubeosis iridis.

## Introduction

Neovascular glaucoma (NVG) is secondary glaucoma that can lead to serious visual impairment. The most important factor in the pathogenesis of NVG is posterior segment ischemia [1]. It has been reported that angiogenic factors produced by a hypoxic retina may facilitate neovascularization of the iris and angular structures, including the trabecular meshwork [2,3]. In the early stages of the disease, neovascularization of the iris and angle (rubeosis iridis) may not affect aqueous humor outflow. However, in advanced stages, as the proliferation of a fibrovascular membrane covers the trabecular meshwork, intraocular pressure (IOP) can increase, leading to NVG development [4,5].

According to population-based studies, the prevalence of NVG ranges from 0.20% to 0.55% [6–8]. This relatively low prevalence makes it difficult to access the etiology of NVG using population-based studies. Therefore, several studies of hospital-based populations have reported the underlying causes of NVG with inconsistent results [9–16]. For instance, in 1974, Hoskins et al [9] reported that diabetic retinopathy (DR; 33%) and retinal vein occlusion (RVO; 28%) were the main causes of NVG among US patients. Brown et al [10] reported that RVO (36.1%) was the most common cause of NVG among US patients, followed by DR (32.2%) and carotid artery occlusion (12.9%). Based on these results, it is generally accepted that DR, RVO, and other diseases, including ocular ischemic syndrome (OIS), account for approximately one-third of the etiologic factors of NVG. Among Saudi Arabian patients, DR (56.1%) was the most common cause of NVG, followed by RVO (26.4%) and chronic retinal detachment (3.6%) [11]. When a Nigerian African population was investigated, RVO was the leading cause (78.7%) of NVG, and DR was responsible for only 1.6% of cases [12]. Recently, Liao et al [16] reported that in China, DR was the leading cause (39.7%) of NVG, followed by RVO (22.9%). These findings suggest that the etiology of NVG may vary among populations. To date, little is known about the etiology of rubeosis iridis among Asian populations, including Korean patients. We hypothesize that the distribution of etiologic factors for rubeosis iridis in Korean patients may differ from those presented in previous studies using different populations. This study was performed to investigate the etiology and clinical features of eyes with rubeosis iridis among Korean patients who visited an eye hospital in Seoul, Korea.

## Materials and Methods

The study protocol was approved by the Institutional Review Board of our institution. As this study is based on retrospective chart review, informed consent from the participants was not required; the Institutional Review Board approved the exemption of informed consent. All procedures conformed to the guidelines of the Declaration of Helsinki. This study involved a retrospective chart review of Korean patients with rubeosis iridis who visited an eye hospital during the period of January 2010 to April 2013. All cases were reviewed for their basic demographic data, including age at the time of diagnosis, sex, medical history, history of ocular surgery, and the most likely cause of rubeosis iridis as documented by the physician. Complete ophthalmic examination, including best-corrected visual acuity (BCVA) measurement using a Snellen visual acuity chart, slit-lamp examination, IOP measurements with a Goldmann applanation tonometer, and fundoscopy were performed on all eyes. Further examinations, including fluorescein angiography, carotid Doppler, and B-scan ultrasonography were performed in selected cases according to the physician’s discretion.

We defined rubeosis iridis as visible blood vessels on the iris surface detected during the slit-lamp examination. IOP was not considered an inclusion or exclusion criterion; thus, the eyes with various IOP levels were included in the study. In subjects with rubeosis iridis in both eyes, we chose the eye with more severe neovascularization for analysis. Some patients had two disorders that can cause rubeosis iridis in a single eye. For instance, in an eye with DR and RVO, both conditions may contribute to the development of rubeosis iridis. In such cases, the condition that induced more severe retinal ischemia based on the disease stage (proliferative DR versus non-proliferative DR; ischemic RVO versus non-ischemic RVO) was selected as the probable cause.

### Statistical analysis

Clinical characteristics, including age, sex, BCVA, IOP at presentation, and the most likely cause of rubeosis iridis were analyzed. Normality of variables was assessed by Kolmogorov-Smirnov tests. Variables with a normal distribution were presented as mean (standard deviation [SD]) with range, and variables without a normal distribution were presented as median (interquartile range). Percentages (%) were used to present categorical variables.

The eyes were subdivided into three groups based on the most likely cause of rubeosis iridis. Age, sex, and IOP at presentation were compared among the groups using Fisher’s exact test for sex distribution and one-way analysis of variance for age and IOP with *post hoc* analysis. A *P*-value < 0.05 was considered statistically significant. Statistical analyses were performed using SPSS version 12.0 (SPSS, Chicago, IL. USA).

## Results

In this study, we enrolled 533 eyes of 533 Korean participants with rubeosis iridis. The mean age of the participants was 57.9 (13.3) years (range, 11–90). Of these patients, 374 (70.2%) were men and 159 (29.8%) were women. Among the 533 subjects, 163 (30.6%) had systemic hypertension, 392 (73.5%) had diabetes, and 128 (24.0%) had both diseases. The mean (SD) IOP recorded at the time of diagnosis was 37.3 (13.6) mmHg (range, 7–70). The BCVA recorded at the time of rubeosis iridis diagnosis ranged from 20/20 to no light perception; median visual acuity was counting fingers (interquartile range, 0.1). The presumed etiologic factors found in the study population are presented in Table 1.

**Table 1:** Presumed etiologic factors associated with rubeosis iridis.

Primary Cause of Rubeosis Iridis	No. of Cases (% of Total)
Diabetic retinopathy	337 (63.2%)
Retinal vein occlusion	101 (18.9%)
Central	85 (15.9%)
Hemispheric	4 (0.8%)
Branch	12 (2.3%)
Ocular ischemic syndrome	24 (4.5%)
Retinal detachment	17 (3.2%)
Uveitis	15 (2.8%)
Miscellaneous	18 (3.4%)
Central retinal artery occlusion	5 (0.9%)
Endophthalmitis	2 (0.5%)
Hypertensive retinopathy	2 (0.4%)
Cilioretinal artery occlusion	1 (0.2%)
Retinal vasculitis	2 (0.4%)
Trauma	6 (1.2%)
Unknown	21 (3.9%)
Total	533 (100%)

### Diabetic Retinopathy

DR was the most common cause of rubeosis iridis, accounting for 337 of 533 cases (63.2%). Among these 337 patients, 241 were men (71.5%) and 96 were women (28.5%). Mean (SD) age among these 337 subjects was 56.5 (12.2) years. At the time of diagnosis, mean (SD) IOP was 37.3 (13.5) mmHg and median visual acuity was 6/200. All 337 cases had proliferative DR. Among all 533 subjects, bilateral rubeosis iridis was found in 32 subjects (6.0%); all of these 32 cases of bilateral rubeosis iridis occurred secondary to DR. Among the cases secondary to DR, bilateral rubeosis iridis accounted for 9.5%.

### Retinal Vein Occlusion

RVO was primarily responsible for the development of rubeosis iridis in 101 of 533 cases (18.9%); central RVO accounted for 85 cases (15.9%), hemispheric RVO accounted for 4 cases (0.8%), and branch RVO accounted for 12 cases (2.3%). Among these 101 cases, 69 patients were men (68.3%) and 32 patients were women (31.7%). The mean (SD) age of these 101 subjects was 61.0 (14.0) years. At the time of diagnosis, mean (SD) IOP of this group was 39.5 (13.4) mmHg and median visual acuity was counting fingers. The mean period from a diagnosis of central RVO to rubeosis iridis diagnosis was 4 months (range, 1–13). Among the 533 eyes, 12 (2.3%) had both DR and RVO; in such cases, the condition at the more severe stage was considered the etiologic disorder.

### Ocular Ischemic Syndrome

Of 533 patients, 24 cases (4.5%) had rubeosis iridis associated with OIS. Among the 24 patients, 19 patients were men (79.2%) and 5 patients were women (20.8%). The mean (SD) age of these 24 subjects was 64.8 (9.7) years. At the time of diagnosis, mean (SD) IOP in this group was 25.8 (12.8) mmHg and median visual acuity was counting fingers. Eight of these cases (33.3%) were confirmed as carotid artery occlusion by carotid Doppler imaging.

### Other causes

In 21 patients (3.9%), no underlying etiologic factor for rubeosis iridis was found due to media opacity or patient unwillingness to cooperate for further evaluation. Rubeosis iridis was found in association with a chronic retinal detachment in 17 cases (3.2%). Fifteen patients (2.8%) had a history of uveitis and no other possible etiology. Five patients (0.9%) had rubeosis iridis following central retinal artery occlusion. Two patients (0.5%) had hypertensive retinopathy, two patients (0.5%) had endophthalmitis, and six patients (1.2%) had a history of ocular trauma without other potential causes of rubeosis iridis. Cilioretinal artery occlusion, as detected by fluorescein angiography, accounted for one case (0.2%), and retinal vasculitis was found in two cases (0.4%).

### Comparison of clinical characteristics according to the etiology

When the clinical characteristics of the eyes with rubeosis iridis associated with DR, RVO, and OIS were compared, age at the time of diagnosis of rubeosis iridis was lower in patients with DR (56.5 years) than in those with RVO (61.0 years) and OIS (64.8 years, *P* < 0.01). IOP of the eyes with DR (37.3 mmHg) and RVO (39.5 mmHg) was higher than that of the eyes with OIS (25.8 mmHg, *P* < 0.01; Table 2). The proportion of the eyes with increased IOP (IOP > 21 mmHg) among the eyes with DR, RVO, and OIS were 89.0%, 88.1%, and 62.5%, respectively (*P* = 0.01).

**Table 2:** Comparison of the clinical characteristics among the eyes with rubeosis iridis associated with diabetic retinopathy (DR), retinal vein occlusion (RVO), and ocular ischemic syndrome (OIS; mean [standard deviation] and range for age and intraocular pressure [IOP], the number of eyes for sex distribution).

	DR (n = 337)	RVO (n = 101)	OIS (n = 24)	P value
Age (years)	56.5 (12.2) (21–87)	61.0 (14.0) (19–89)	64.8 (9.7) (44–88)	< 0.001*
Sex (F:M)	96:241	32:69	5:19	0.557^†^
IOP (mmHg)	37.3 (13.5) (7–70)	39.5 (13.4) (12–69)	25.8 (12.8) (8–46)	< 0.001*

*One-way analysis of variance with Bonferroni *post hoc* analysis

DR group < RVO group/OIS group for age, DR group/RVO group > OIS group for IOP

^†^Fisher’s exact test

## Discussion

Among Korean patients who visited an eye hospital in Seoul, the most common cause of rubeosis iridis was DR (63.2%), followed by RVO (18.9%) and OIS (4.5%). When the clinical characteristics among the eyes with rubeosis iridis associated with DR, RVO, and OIS were compared, age at the time of diagnosis of rubeosis iridis was lower in patients with DR than in those with RVO and OIS. IOP of the eyes with DR and RVO was higher than that of the eyes with OIS. To the best of our knowledge, this is the only study reporting the etiology and clinical features of rubeosis iridis in a Korean population, which is important to help stratify high-risk patients in order to diagnose this condition earlier.

The results of this investigation are in line with those of previous studies on US patients, in which DR and RVO were the leading causes of NVG [9,10]. However, compared to previous reports, in our series, DR was more common and RVO less common as the cause of rubeosis iridis (Table 3). In US-based studies, DR and RVO were primarily responsible for the development of NVG in approximately one-third of cases, whereas in our study population, DR accounted for approximately two-thirds of rubeosis iridis cases, and RVO was responsible for only 18.9% of cases. This inconsistency may be explained in part by differences in the prevalence or clinical characteristics of DR, RVO, or systemic disorders, including diabetes, hypertension, dyslipidemia, and atherosclerosis, which are identified risk factors for DR and RVO in the studied populations [17,18].

**Table 3:** Comparison of etiologic factors associated with rubeosis iridis among different study populations.

Author (year)	Hoskins (1974)	Brown (1984)	Al-Shamsi (2009)	Liao (2016)	Present study (2016)
Region of study	US	US	Saudi	China	Korea
Number of patients	100	208	337	310	533
Main causes					
Diabetic retinopathy	33%	32.2%	56.1%	39.7%	63.2%
Retinal vein occlusion	28%	36.1%	26.4%	22.9%	18.9%
Ocular ischemic syndrome	8%	12.9%	1.8%	2.3%	4.5%
Retinal detachment	3%	1.5%	3.6%	5.5%	3.2%
Uveitis	11%	1.5%	0.5%	1.9%	2.8%
Other causes	14%	11.4%	3.6%	8.7%	3.4%
Unknown	3%	4.4%	8.0%	19.0%	3.9%

When interpreting the previous study results from US patients, it should be noted that these studies were published decades ago: 1974 [9] and 1984 [10]. Since that time, although many studies have reported NVG treatment outcomes, little has been reported about the etiologic factors of rubeosis iridis or NVG among US patients. One may speculate that etiologic factors for a condition may change over time within a given population. Thus, we believe that it is necessary to revisit the current etiologic factors of rubeosis iridis among US patients.

Regarding the etiology of NVG among Asian populations, Kiddee et al [13] studied a Thai population and reported that the most common etiology of NVG was RVO (47%), followed by DR (42%) and OIS (5%). This result contrasts our findings. Liao et al [16] reported that in China, DR (39.7%) was the most common cause of NVG, followed by RVO (22.9%) and unknown cause (19.0%). Although, the first and second most common causes were similar to our results, the proportion of DR in that study (39.7%) was lower than that of the present study (63.2%). To date, little is known about the effect of ethnicity on etiology of rubeosis iridis. Further studies investigating the etiology of NVG among various populations are necessary.

In previous studies, included subjects had NVG and high IOP [9–13]. However, on some occasions, rubeotic eyes may be normotensive in early stages or even hypotensive because of reduced aqueous humor production [19]. Thus, the enrollment of only rubeotic eyes with high IOP may lead to a selection bias. Therefore, our series included subjects with rubeosis iridis regardless of IOP level to not exclude normotensive eyes with rubeosis iridis. When only eyes with high IOP (>21 mmHg) were assessed separately, the main causes of rubeosis iridis were similar to the results from the group overall; the most common cause of rubeosis iridis was DR (64.4%), followed by RVO (19.1%) and OIS (3.2%). Therefore, we believe that the differences in inclusion criteria among studies did not play a major role in the differing results between these studies.

In our study population, patients with rubeosis iridis primarily caused by DR were younger than patients with other underlying causes; mean ages at the time of NVG development in patients with DR, RVO, and OIS were 56.5, 61.0, and 64.8 years, respectively. Previous studies also reported that eyes with NVG caused by DR were associated with younger age than eyes with NVG due to other causes, including RVO or OIS [9–11,20]. Differences in onset age, the impact of retinal vessels, or ocular blood flow among DR, RVO, and OIS may have contributed to this finding. In the present study, the type of diabetes and the presence of coagulation disorders were not analyzed because such data are not usually described in medical records. Given the lower ranges of age in the DR (21 years) and RVO groups (19 years) than in the OIS group (44 years), patients with type 1 diabetes in the DR group or young patients with coagulation disorders in the RVO group may contribute in part to the difference in age distribution among the groups.

Another feature of rubeosis secondary to DR was a higher prevalence of bilateral involvement than that in rubeosis due to other conditions; bilateral rubeosis iridis was found in 6% of eyes, and all had rubeosis due to DR. Brown et al [10] reported that bilateral NVG was found in 12% of the eyes studied, and 96% of the cases were secondary to DR, which is in agreement with the results of the present study. Al-Shamsi et al [11] reported that the incidence of DR in bilateral NVG was 88%, whereas it was only 8% in RVO cases. In the report by Liao et al., 8.4% eyes had bilateral NVG and among these eyes, 76.2% had DR [16]. These findings may be explained by the higher incidence of bilateral involvement and relative symmetry of DR compared to RVO or other conditions [21]. The greater likelihood of bilateral rubeosis iridis in eyes with DR underscores the importance of cautiously exploring the ocular health of the fellow eye.

In the present study, the mean time from a central RVO diagnosis to a rubeosis iridis diagnosis was 4 months (range, 1–13). Previous studies reported that over 80% of NVG cases were identified within the first 6 months after RVO development [22]. Therefore, a detailed inspection of the iris and angle must be performed during this period.

Rubeotic eyes with OIS showed relatively lower IOP than eyes with DR or RVO. Among eyes with OIS, approximately one-third of the cases did not have elevated IOP. Previous studies reported that increased IOP and NVG are noted in only 50% of patients with OIS, and ocular hypotony may also be observed in spite of fibrovascular tissue, secondary to neovascularization, obstructing the angle [23,24]. This finding may be due to ciliary body ischemia and reduced production of aqueous humor [23,24].

A limitation of our study is that subjects were studied retrospectively and that the diagnosis of the systemic disease was based only on a review of medical history. In addition, there was a lack of detailed data regarding systemic disorders, such as diabetes type and HbA1c levels, dyslipidemia, or atherosclerosis. We only enrolled patients who visited an eye hospital in Seoul, Korea; therefore, our population may not represent the general Korean population. To ensure the validity of our study results, other studies from various Korean populations are needed. Moreover, in the present study, rubeosis iridis was defined as visible vessels only on the iris surface. Given that abnormal vessels may also arise in the anterior chamber angle earlier than they appear on the iris surface, the ideal method for assessing rubeosis iridis etiology is by using gonioscopy in all patients with DR, RVO, OIS, uveitis, or other retinal disorders. However, in the clinic, it is difficult to perform gonioscopy in all patients. Among the present study population, those who visited the glaucoma clinic underwent gonioscopy, whereas the majority of patients who only visited the retina hospital of our institution did not undergo gonioscopy. Therefore, in the present study, the presence of rubeosis iridis was confined to visible vessels on iris surface; gonioscopic findings were not included. This may have affected the study results. If we performed gonioscopy in all patients, more eyes with new vessels would have undoubtedly been found. However, its effect on the distribution of etiology of rubeosis iridis remains unknown. From a clinical perspective, the course of the disease, including IOP control by medication or surgery, visual acuity change, and the effect of etiology on the clinical course would also provide valuable information. As this study aimed to investigate the etiology and clinical characteristics, the longitudinal course and treatment outcomes of patients with rubeosis iridis will be presented in subsequent reports.

In conclusion, DR was a leading cause of rubeosis iridis followed by RVO and OIS in this Korean population who visited an eye hospital in Seoul, Korea. This finding may be useful when assessing the cause of rubeosis iridis in Korean patients.
